# Improving eating disorders "mental health literacy" in young people: a preliminary evaluation

**DOI:** 10.1186/2050-2974-1-S1-O37

**Published:** 2013-11-14

**Authors:** Kassandra Gratwick-Sarll, Caroline Bentley, Jonathan Mond

**Affiliations:** 1 The Australian National University, Australia; 2 The University of Canberra, Australia

## Background

We sought to provide preliminary evidence for the effectiveness of an intervention designed to improve eating disorders mental health literacy (ED-MHL) in young people.

## Methods

Participants were 177 young men and women recruited from a University campus. Key aspects of ED-MHL, including awareness and understanding of the nature and treatment of eating disorder (ED) behaviour, the importance of early, appropriate help-seeking and barriers to treatment such as poor insight and attitudes likely to be conducive to stigma, were assessed by means of self-report questionnaire before and immediately after the intervention. The intervention comprised a single, three-hour workshop, developed by Hart and colleagues, designed to address a broad range of issues relating to the nature and treatment of EDs.

## Results

Preliminary analysis of pre-post data indicated benefits in terms of improved awareness and understanding of ED behaviour and its treatment and reduced stigma. Results of a three month follow-up analysis, which will be reported at the conference, will establish whether these changes in knowledge are (i) sustained and (ii) accompanied by change in behaviours such as the promotion of early, appropriate help-seeking among individuals with symptoms.

## Conclusions

A brief psychoeducational intervention appears to be beneficial in improving ED-MHL in young people.

This abstract was presented in the **Prevention** stream of the 2013 ANZAED Conference.

